# The Cinderella syndrome: why do malaria-infected cells burst at midnight?

**DOI:** 10.1016/j.pt.2012.10.006

**Published:** 2013-01

**Authors:** Nicole Mideo, Sarah E. Reece, Adrian L. Smith, C. Jessica E. Metcalf

**Affiliations:** 1 Center for Infectious Disease Dynamics, The Pennsylvania State University, University Park, PA 16802, USA; 2 Centre for Immunity, Infection, and Evolution, University of Edinburgh, Edinburgh, EH9 3JT, UK; 3 Institutes of Evolutionary Biology and Immunology and Infection Research, University of Edinburgh, Edinburgh, EH9 3JT, UK; 4 Department of Zoology, University of Oxford, Oxford, OX1 3PS, UK

**Keywords:** *Plasmodium*, synchronicity, circadian rhythms, evolution

## Abstract

An interesting quirk of many malaria infections is that all parasites within a host – millions of them – progress through their cell cycle synchronously. This surprising coordination has long been recognized, yet there is little understanding of what controls it or why it has evolved. Interestingly, the conventional explanation for coordinated development in other parasite species does not seem to apply here. We argue that for malaria parasites, a critical question has yet to be answered: is the coordination due to parasites bursting at the same time or at a particular time? We explicitly delineate these fundamentally different scenarios, possible underlying mechanistic explanations and evolutionary drivers, and discuss the existing corroborating data and key evidence needed to solve this evolutionary mystery.

“…*sa marraine lui recommanda sur toutes choses de ne pas passer minuit, l’avertissant que si elle demeurait au bal un moment davantage, son carrosse redeviendrait citrouille*…”

“*Her godmother bade her not to stay beyond midnight whatever happened, warning her that if she remained at the ball a moment longer, her coach would again become a pumpkin*” – Charles Perrault

## Coordinated malaria parasites

**Figure 1:**
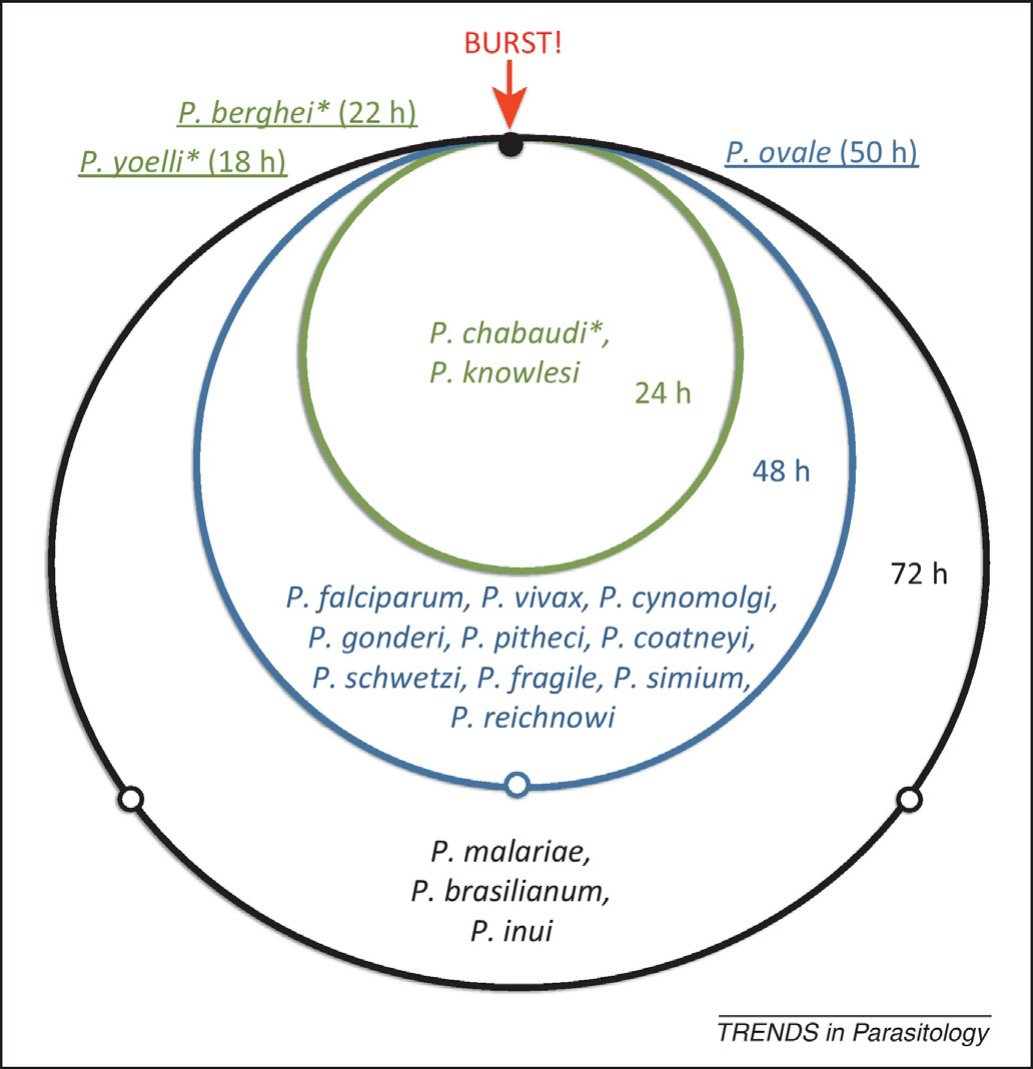
The diversity of mammalian *Plasmodium* cycles. Circle length indicates cell cycle length; small open points show the end of one 24-h period. Species names inside the inner (green) circle have 24-h cycles, species names inside the middle (blue) circle have 48-h cycles, and species in the outer (black) circle have 72-h cycles. Species infecting rodents are marked with an asterisk (*); all other species infect primates. Species with non-24-h cycles are underlined and shown at the appropriate point on their cycle. (*Plasmodium berghei* and *Plasmodium yoelii* are the only species thought to develop asynchronously.) Cycle times are assembled from 34, 35, 36, 37, 38.

For most malaria parasite species, the parasite cell cycle within a host is precisely coordinated – malaria parasites invade host red blood cells (RBCs), replicate asexually, and then release the next cohort of parasites in a burst that is synchronized across all parasites within the infection. Intriguingly, this synchronized bursting appears to occur at a particular time: *Plasmodium chabaudi* (rodent malaria) parasites, for instance, tend to synchronize bursting around midnight [1] (thus inspiring our title, although it is important to point out that other *Plasmodium* species burst at different times of the day/night). Although cell cycle duration varies across malaria parasite species, it is generally a multiple of 24 h (Figure 1). Indeed, the periodicity of fever that follows the simultaneous bursting of RBCs at the end of the cell cycle was once used as a diagnostic tool (e.g., the human parasites *Plasmodium falciparum* and *Plasmodium malariae* have 48 and 72 h cycles, respectively, leading to ‘tertian’ or ‘quartan’ fevers [2]).

Despite an early interest in evolutionary explanations for the coordination of malaria parasites (e.g., [3]), work in recent years has focused on identifying proximate mechanisms for such rhythms. The 24-h cell cycles (or multiples thereof) are suggestive of a circadian basis, and the search continues for homologs of clock genes in the parasite genome [4] and host circadian cues that could influence parasite cell cycles (e.g., melatonin [5]). By uncovering these mechanisms it may be possible to manipulate parasite schedules as a form of control. Interestingly, changes to parasite rhythms have been implicated in resistance to current front-line antimalarial drugs (artemisinin derivatives); parasites are thought to enter a quiescent state, delaying their development until the activity of drugs in their host has diminished (e.g., 6, 7). This highlights the need to understand why parasites have a precisely timed schedule at all, what the evolutionary constraints are on their developmental schedule, and what the consequences would be of targeting this fundamental part of malaria parasite biology.

## Circadian parasites?

Circadian rhythms are endogenous patterns that persistently occur approximately every 24 h [8]. These rhythms, entrained according to some external stimulus such as a photoperiod, are a consequence of organisms needing to predict changes in their environment, for example, to adopt appropriate activities for day and night [9]. Although coping with such periodic changes is a fundamental problem for organisms across the tree of life, establishing an adaptive basis for periodicity in behavior or physiological processes is not straightforward 10, 11. More difficult still is explaining why parasites that mostly – or exclusively – live within the bodies of other organisms should evolve a circadian rhythm, yet malaria parasites are not unique in this respect.

The conventional argument for the evolution of periodicity in parasites is that it optimizes the production of transmissible parasite forms given the diurnal rhythms of the environment. For example, coccidian parasites of the genus *Isospora* are infective to new hosts only after transmissible forms (immature oocysts) are excreted and have undergone further development in the external environment. These transmissible forms tend to emerge later in the day, apparently avoiding environmental conditions that are unfavorable to survival and development 12, 13, 14. Early work on malaria parasites emphasized similar evolutionary thinking, and the timing of foraging activity of mosquitoes (the malaria vector) was argued to be the key selection pressure driving cell cycle coordination [3]. However, this ‘Hawking hypothesis’ lacks logical coherence and empirical validation (Box 1). The evolutionary drivers of the coordination of malaria parasite cell cycles therefore remain mysterious. Below we offer a conceptual framework for studying this phenomenon and evaluate other evolutionary hypotheses in the light of emerging insight into the mechanisms involved in generating rhythms in malaria parasites.

Box 1 **Malaria transmission and the Hawking hypothesis**

Transmission of malaria to mosquitoes occurs via specialized parasite forms called gametocytes that are produced by a small subset of infected RBCs. The ‘Hawking hypothesis’ suggests that the production of gametocytes is coordinated so that they reach maturity and maximum infectiousness when mosquitoes feed 3, 39, 40, but tests of its underlying assumptions have been equivocal. First, the hypothesis assumes that synchronized maturation of gametocytes requires synchronized bursting of all infected RBCs. Because gametocyte maturation can take as long as 15 days (for *P. falciparum* [41]), even slight differences between gametocytes in maturation rates could accumulate to result in differences of many hours by the time maturation is complete. Second, the hypothesis assumes that mature gametocytes circulate in the bloodstream for less than a day (or only one bout of mosquito foraging). Although estimates suggest that the gametocytes of a few malaria species have circulation times on the order of hours 42, 43, estimates for *P. falciparum* span several days (reviewed in 41, 44). Finally, it assumes that maximum infectiousness coincides with peak mosquito biting activity, which is a pattern that most studies have failed to find (e.g., 45, 46, 47). It has been argued that data on infectivity are too sparse to confidently reject the Hawking hypothesis [48]. We agree that more detailed data are required to fully understand any rhythms in gametocyte and vector biology, but argue that the bulk of evidence against the Hawking hypothesis is probably insurmountable.

## The what and why of coordination

From our perspective, what has been lacking from studies of malaria parasite cell cycles is an explicit recognition that the observed coordination could be the effect of selection for either synchrony or timing (see Figure I in Box 2); most evolutionary hypotheses have implicitly assumed that parasites gain an evolutionary advantage from one of these traits and ignored the other. For simplicity, we refer to synchrony and timing as ‘traits’ but stress that in reality these phenotypes are the outcome of several underlying traits. Alternatively, coordination may provide no evolutionary advantage to parasites, may actually benefit hosts, and may be entirely under the control of hosts. Framing the evolution of coordination in this way reveals five possible ‘selective scenarios’, outlined below and detailed in Table 1.

(i) Synchrony of cell cycles is an adaptation of malaria parasites that enhances their fitness. For example, synchrony could provide parasites with safety in numbers from immune attack. To coordinate this synchrony, parasites could either communicate directly (e.g., via a development communicating mechanism [15]), they could have their own internal clock, or they could use timing cues from host circadian rhythms 2, 5. In the first case, synchrony and timing would not be linked and so synchronized parasites in different hosts may burst at different times of day. In the latter two cases, because parasites are using time cues to achieve synchrony, bursting at a precise time would likely be a byproduct of selection for synchrony (but does not provide an additional advantage to parasites).
(ii) The timing of bursting is an adaptation of malaria parasites (i.e., infected RBCs bursting at a particular time of the day/night maximizes fitness). For example, timing could allow parasites to avoid a circadian release of parasite killing immune responses or match the appearance of essential resources. Assuming a particular schedule offers the same benefit to all parasites in an infection, synchrony would be a byproduct of the evolution of timing because all parasites would be on the same schedule. Parasites could set the timing of cell cycle development either indirectly by using cues from the host circadian rhythm or directly using their own internal ‘clock’.
(iii) Synchrony and timing of cell cycles are both adaptations of malaria parasites and may be selected for through different processes. In other words, each trait offers a distinct fitness advantage, that is, some combination of (i) and (ii).
(iv) Neither synchrony nor timing of cell cycles is an adaptation of malaria parasites. Instead, the coordination of cell cycles offers some advantage to hosts. For example, concentrating particular parasite cell cycle stages to a particular time of day could maximize the efficacy of immune factors with circadian rhythms [16] or could limit invasion success by maximizing competition between parasites for host RBCs.
(v) Neither synchrony nor timing of cell cycles is an adaptation of malaria parasites or hosts. Instead, parasites are passively traversing their life cycle and the cell cycle pattern is constrained by physiological or physical features of either, or both, hosts and parasites (see Figure II in Box 2). Under this scenario, the coordination of parasite development may be neutral or costly to either hosts or parasites.

Box 2 **The malaria cell cycle and its potential constraints**

Malaria parasites replicate asexually within host RBCs before bursting and releasing the progeny parasites (merozoites), each with the capacity to invade other RBCs and begin a new cycle of replication. Explaining the evolutionary significance of the observed periodicity of malaria infections requires considering the importance of two distinct traits (Figure I).

**Figure I:**
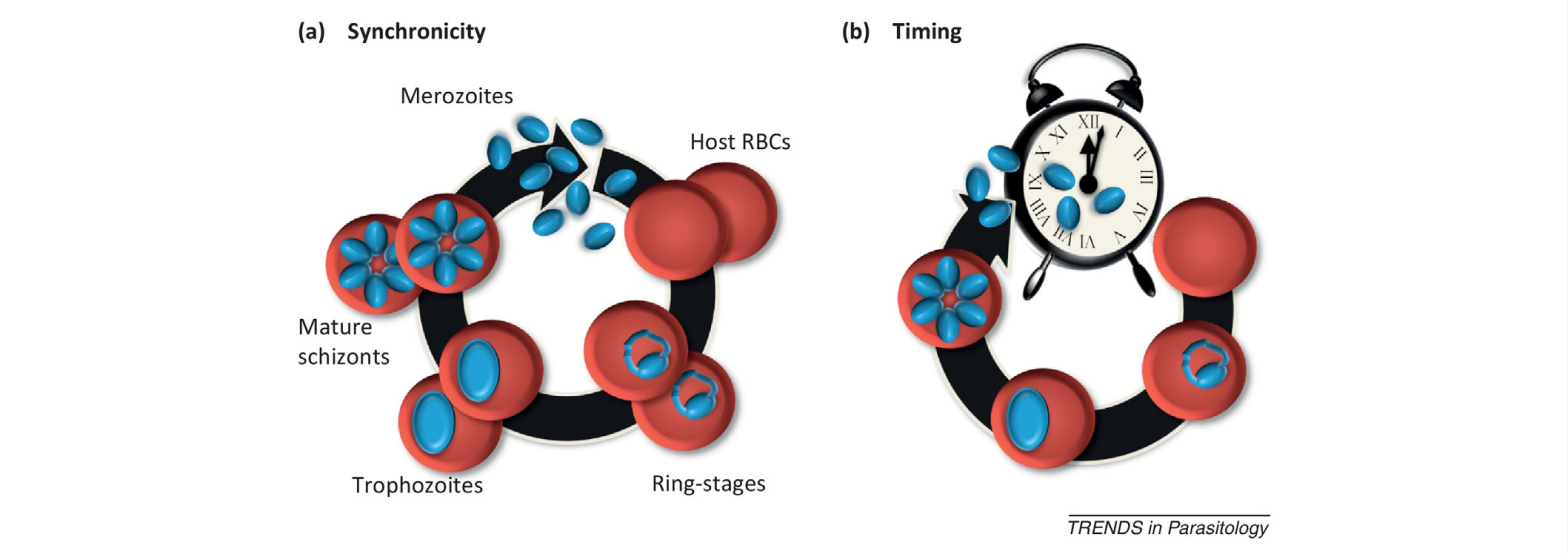
Two developmental ‘traits’. **(a)** There may be an advantage to all parasites within an infection progressing through the cell cycle in synchrony. **(b)** Alternatively, there may be an advantage to timing, where transitions to different developmental stages occur at specific times of day. Of course, both traits may be advantageous independently or may be correlated, for example, if parasites use a host circadian cue as a signal to coordinate bursting.

Figure II illustrates the potential constraints on malaria parasite cell cycles.

**Figure II:**
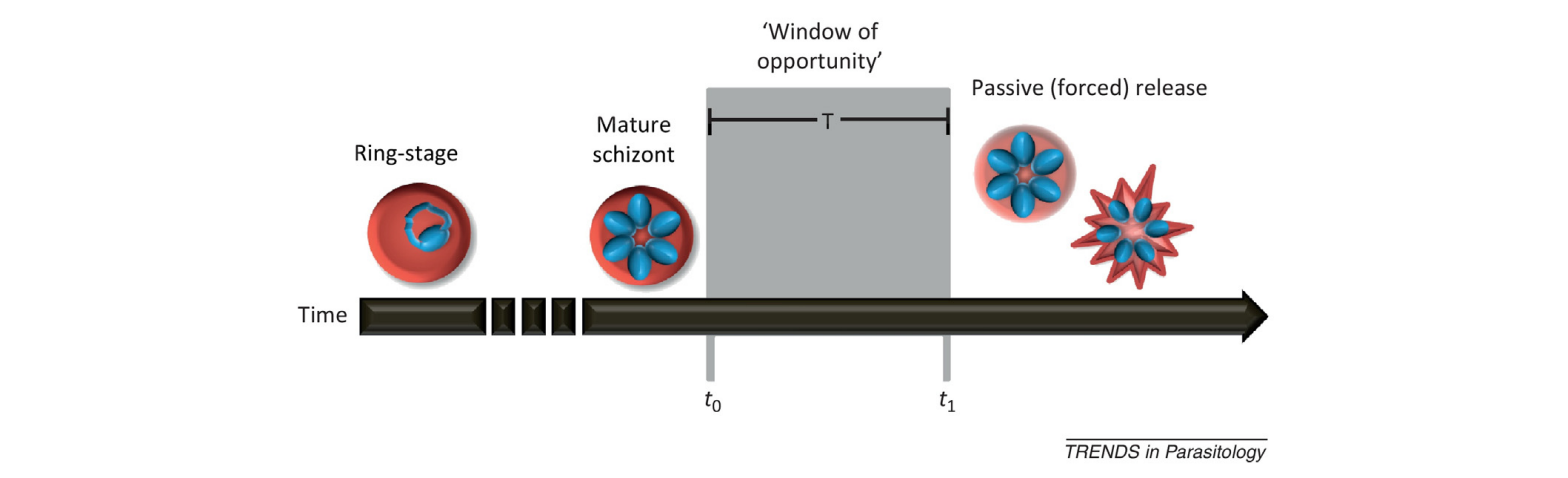
Physical and physiological constraints on cell cycles. The physical processes of RBC invasion and parasite replication take time, meaning that merozoite release cannot happen before a time, *t*_0_. Although malaria parasites complete all nuclear divisions prior to cellular division [50] (making the process more efficient), increasing the number of merozoites produced could increase the time required until a mature schizont is ready to burst. *Plasmodium* species that produce different numbers of merozoites may therefore have different values of *t*_0_. At the other end of the spectrum, once the nuclear divisions have been completed, there may be an upper limit on bursting time, *t*_1_, as parasites may be constrained by passive processes that cause bursting, through deterioration of the infected RBC or bursting under osmotic stress [51]. The difference between times *t*_0_ and *t*_1_ define the window of opportunity during which parasites can burst from infected RBCs.

Recent experimental work suggests that infected RBCs are, indeed, not bursting at the earliest possible opportunity, *t*_0_, [49]. Using an *in vitro* approach, Grüring *et al*. [49] demonstrate that merozoite formation and the maturation of schizonts are completed several hours before bursting. Although not conclusive evidence, because it is not clear whether merozoites are finished developing at the submicroscopic level, this suggests that schizonts are not bursting as soon as possible and that they are waiting for some cue that either allows them to synchronize [scenario (i)], forces them to synchronize [scenario (iv)], or signals optimal timing of bursting [scenarios (ii) or (iii)].

**Table 1:** Putative evolutionary explanations^a^ for coordination as an adaptation of malaria parasites

Ultimate explanation	Selective scenario^b^	Key evidence required to evaluate hypotheses
**Transmission**		
*1. Biting vector ecology*. Bursting is timed to maximize transmissible gametocyte stages present when vectors are biting (e.g., 3, 39, 40).	(ii)	• Does gametocyte availability and/or infectiousness match the timing of vector blood-feeding behavior? • Does the timing match across different interacting vector and parasite species? • Does synchronicity break down during periods when few gametocytes are produced?
**Immunity**		
*2. Circadian immune effectors.* Immune effectors (IEs) experience a circadian cycle (e.g., 29, 52, 53, 54, 55) and coordinated cell cycles avoid exposing the most vulnerable parasite stages to these IEs.	(ii)	• Do circadian rhythms in IEs translate to differences in parasite killing capacity? • How susceptible are different parasite stages to these IEs? • Is there temporal mismatch between the presence of a parasite stage and the most effective IEs? • Do cell cycles become more or less coordinated over the course of infections as the efficacy of IEs changes?
3. *Lagged immune response.* A delay between immune recognition of a particular parasite stage and immune action on that stage by innate immunity will favor synchrony [22].	(i)	• Is there temporal mismatch between the presence of a parasite stage and its most damaging IEs? • Do cell cycles become more or less coordinated over the course of infections as the efficacy of IEs changes?
*4. Dilution effect*. Innate IEs are overwhelmed by the numbers of parasites released (analogous to masting by trees, herding by animals).	(i)	• Does per capita mortality of merozoites decline with per capita density [56]? • Does any density dependence of merozoite mortality vanish in immune depleted mice?
**Resource supply**		
*5. Circadian resource availability.* Circadian rhythms in RBC release have been directly observed 57, 58 and inferred 59, 60; bursting coincides with appearance of new RBCs to maximize efficiency of resource acquisition.	(ii)	• When RBCs are not limiting, do reticulocyte- (young RBC) preferring species and mature RBC-preferring species show different timing of bursting? Only reticulocyte-preferring species would be predicted to burst in step with RBC release because mature RBCs are always available. • Are the costs of jet-lagging parasites greater when RBCs are limiting than when abundant?
*6. Circadian resource quality.* Circadian cycles in RBC biochemistry mean that RBC invasion is most successful at a particular time of day, or provide the best habitat for replication only at a particular time (e.g., redox status of RBCs follows a circadian rhythm [61]).	(ii)	• Are less circadian parasites (with cycles that are not multiples of 24 h) able to invade or replicate their DNA in RBCs throughout the day? • Does the success of establishing a new infection in *in vivo* experiments depend on the time of day of inoculation?
**Other within-host environmental conditions**		
*7. Turbulence.* Blood pressure follows a circadian rhythm [62] and bursting occurs when turbulence in the blood stream is at some optimum.	(ii)	• Is timing different for parasites that infect nocturnal versus diurnal host species? • *In vitro*, is schizont survival and success of RBC invasion affected by sample shaking? • If turbulence is a function of heart rate, does cycle length (24, 48, 72 h) correlate with host size (a proxy for heart rate)?
8. *Splenic clearance.* Cytoadherence and rosetting, the results of shuttling proteins to the surface of infected RBCs, help prevent parasite clearance by the spleen [63]. These evasion strategies are most effective if all parasites are at the same developmental stage (i.e., expressing sticky surface proteins at the same time).	(i)	• Does rosetting occur in asynchronous parasite species? • Can asynchrony be selected for by passaging parasites through splenectomized hosts?

a Non-mutually exclusive.

b The selective scenario indicates which ‘trait’ is under selection: (i) denotes a direct advantage to synchrony and (ii) denotes a direct advantage to timing.

With these scenarios explicitly laid out, the next challenge is to determine which gave rise to the patterns of coordination observed in malaria parasite cell cycles. An explanation that does not involve a fitness benefit to parasites [scenario (iv) or (v)], but instead is a coincidental outcome of within-host interactions is appealing in its relative simplicity: an array of host-dependent processes could produce patterns that appear to be parasite coordination. For example, Kwiatkowski and Nowak [17] used a mathematical model to show that if merozoite release triggers fever or fast acting immune effector mechanisms that kill any merozoites that are released later (or other infected RBC stages, e.g., trophozoites), the observed pattern of parasites in the bloodstream would look like synchrony. Rather than being an adaptation of the parasites however, this would be simply the footprint of host responses, which could be beneficial to the hosts [scenario (iv)] [18]. This sort of mechanism could explain why synchrony breaks down in culture conditions, where host rhythms are absent [19].

However, parasites detect and respond to host circadian cues *in vitro* (e.g., [5], reviewed in [20]), which suggests that, rather than being an artifact of stage-selective parasite killing, parasites are coordinating themselves [scenarios (i)–(iii)]. Furthermore, the same mouse strains are often used in *in vivo* experimental infections with synchronous and asynchronous species of rodent malaria parasites, suggesting that host factors alone cannot be driving coordination. Finally, recent work with one of these experimental systems provides the clearest evidence yet that coordination offers an advantage to parasites. In experiments where synchronous *P. chabaudi* parasites were temporally mismatched to the circadian rhythm of their mouse host, ‘jet-lagged’ parasites suffered costs (i.e., reduced production of replication and transmission stages) compared with parasites in circadian rhythm matched hosts [21]. Given that parasites do worse when they are jet-lagged, their natural coordination probably does not benefit the host [precluding scenario (iv)]. Instead, a compelling explanation for these results is that parasites are using a host-generated cue to schedule their bursting, and jet-lagged parasites pay a cost because they receive this cue at an inappropriate time. What remains unclear is whether matching the host circadian rhythm confers an advantage through timing alone [scenario (ii)], or both timing and synchrony [scenario (iii)] because jet-lagged parasites also became less synchronous (A. O’Donnell, unpublished data).

## Synchronizing against immunity

Although coordinated cell cycles may help parasites cope with a circadian resource supply or other environmental challenges (Table 1), in our view, the most persuasive hypotheses for an adaptive basis to synchrony or timing tend to involve strategies for dealing with the immune response of the host. Synchrony alone could provide a benefit to parasites by allowing them to either evade immunity or overwhelm it. Rouzine and McKenzie [22] show mathematically that if there is a temporal separation between the parasite stage that induces an immune response and the stage that is the target of that immune response, synchrony of developing parasites enables immune evasion. This would be plausible for immune responses with short-lived effectiveness. For example, the fever that follows a coordinated burst creates an environment of elevated inflammatory cytokines and reactive oxygen/nitrogen species that is unfavorable to parasite growth [23] and reduces gametocyte viability/infectivity 24, 25, 26. With synchrony, parasites could concentrate this stress at a time when they are not replicating, and/or minimize the time when transmission to mosquitoes is blocked.

Alternatively, synchrony could allow bursting parasites to overwhelm immunity through sheer force of numbers 16, 18, 21, 27. If the strength of a short-lived immune response is proportional to the insult or stimulus (as might be seen with activation of macrophages after exposure to Toll-like receptor agonists), then the advantage to synchrony appears rather tautological – a coordinated burst is needed to overcome an immune response that is elicited by a coordinated burst. However, this apparent logical conundrum is not insurmountable. Even with a trade-off between immune activation and safety in numbers, there could be a merozoite density over which the benefit outweighs the cost. This is analogous to the ‘dilution–attraction’ trade-off in predator–prey ecology: herding behavior provides safety in numbers, but larger herds may be more likely to attract predators (e.g., [28]). Which of these two effects dominates depends on the precise relationships between herd numbers and both dilution and attraction. Quantifying these sorts of relationships for malaria (i.e., those between merozoite density and both immune activation and immune escape) is no trivial task, because it would be hard to tease the effects apart experimentally. However, without such data, it is inappropriate to rule out this hypothesis based upon intuition alone.

## Timing against immunity

The previous section describes cases where coordination of malaria parasite cell cycles is the result of selection favoring synchrony to overcome host immune responses. The fact that this coordination appears precisely timed could be a simple byproduct of selection for synchrony, especially if synchrony was orchestrated by co-opting a host circadian cue. However, timing itself may offer advantages in the battle against host immune responses. If host immune activity is circadian, then timing could facilitate immune evasion. Circadian rhythms have recently been documented in some arms of immunity [29] that are proving important for the control of malaria infections [30]. However, to show that parasites are scheduling their cell cycle to avoid being vulnerable during peak immune activity, one would have to: demonstrate that peak expression of immunity genes translates to increased parasite killing potential, determine which parasite developmental stages are most vulnerable to immune attack, and show that cell cycles are scheduled so that those stages are not reached during peak immune activity. Because the timing of malaria parasite cell cycles seems, in part, to be determined by host species (e.g., diurnal or nocturnal hosts), it would also be important to know if the same immune mechanisms are important across host taxa and if patterns of immune activity vary accordingly. Although avoiding immunity is a compelling explanation for timing of malaria parasite cell cycles, the empirical evidence has yet to make the case (or be collected).

## Concluding remarks

Explaining cell cycle coordination in malaria parasites will require answering some of the key questions that stem from the different hypotheses outlined in Table 1. Perhaps even before those questions are addressed however, the dearth of within-host natural history data on malaria parasite schedules should be tackled. Relatively few studies have specifically sought to measure variation in cell cycle durations, timing, or synchrony (e.g., 31, 32). The predictions and reasoning developed here assumes that all published data are correct. However, many of the key data exist as point estimates from a very narrow range of circumstances and combinations of host and parasite strains. Our view is that there is real value in improving the accuracy of these estimates and quantifying any natural variation. The literature on circadian rhythms will help clarify the type and quality of data required [4]. For instance, emphasis should be placed on measuring infections at more than one time point per cell cycle, with a resolution sufficient to determine the true extent of coordination, because varying degrees of synchrony can result in the same patterns that differ only quantitatively [33].

Coordination is not well understood for any malaria parasite species and it is possible that broad statements about the evolution of cell cycle coordination cannot actually be made [4]. Indeed, the evolutionary and mechanistic drivers of coordination could vary across species, genotypes, and even during infections. However, a better understanding of the causes and consequences of coordination in any scenario could help shed light on further complexities and open questions (Box 3). From our vantage point, this evolutionary mystery can only be solved with increasingly rigorous collection of evidence as well as a clearly defined evolutionary and ecological framework with which to evaluate that evidence.

Box 3 **Outstanding questions**

• How ‘synchronous’ are synchronous species, that is, what degree of variance in burst time is observed within an infection? How different are asynchronous species in this respect?
• How and why do co-infecting species display different degrees of synchrony (e.g., [34])? In other words, why are some parasites vulnerable without coordination, whereas some parasites can apparently tolerate asynchrony?
• Do species with 48-h or 72-h cycles have to withstand one or two rounds of exposure to cues that make 24-h cell cycle species burst? And if so, how? Or does their existence provide evidence that host circadian cues cannot be scheduling parasite cell cycles?
• Do species with cycle lengths that are not multiples of 24 h reliably reproduce those cycles *in vivo* throughout infections?
• Does the parasite cell cycle length in different *Plasmodium* species correlate with particular aspects of parasite and/or host biology? For example, do parasites with 48-h and 72-h cycles produce more merozoites? (We could not find any convincing evidence of this across species, although the data are sparse. Within species, genotypes with longer cycle lengths do not necessarily produce more merozoites [31].) Do species with longer cell cycles produce more robust or infective merozoites?
• Does the timing of bursting in different *Plasmodium* species correlate with particular aspects of parasite and/or host biology? For example, are parasites that infect nocturnal hosts more likely to burst at midnight? (Again, the data are sparse, but we could find no clear pattern.)
• Does the variation in cycle length within a single malaria species demonstrated in recent work 31, 32 imply that different cycle lengths can coexist?
• What are the fitness consequences – for both hosts and parasites – of the variation observed in cycle length across genotypes (which would result in differences in timing) and within genotypes (which indicates variation in synchrony)? Further work quantifying variation and its fitness effects with an *in vivo* model malaria system is needed to provide insight on whether synchronicity and/or timing are adaptations of parasites or hosts.
• Do the *P. falciparum* genotypes with non-48-h cycles *in vitro* [32] keep to those cycles *in vivo*? Are such phenotypes common in nature and, if so, do they suggest that timing is not important for parasites?
